# Is Breastfeeding an Effective Approach to Reduce Metabolic Risk After GDM in Mothers and Infants?

**DOI:** 10.3390/jcm14093065

**Published:** 2025-04-29

**Authors:** Tiziana Filardi, Enrico Bleve, Stefania Gorini, Massimiliano Caprio, Susanna Morano

**Affiliations:** 1 Department for the Promotion of Human Sciences and Quality of Life, San Raffaele Roma Open University, Via di Val Cannuta, 247, 00166 Rome, Italy; stefania.gorini@uniroma5.it (S.G.); massimiliano.caprio@uniroma5.it (M.C.); 2 Department of Experimental Medicine, “Sapienza” University, Viale Regina Elena 324, 00161 Rome, Italy; enrico.bleve@uniroma1.it (E.B.); susanna.morano@uniroma1.it (S.M.); 3 Laboratory of Cardiovascular Endocrinology, IRCCS San Raffaele, Via di Val Cannuta, 247, 00166 Rome, Italy

**Keywords:** gestational diabetes mellitus, GDM, breastfeeding, lactation, breast milk, type 2 diabetes, postpartum glucose, metabolic health, growth, childhood obesity

## Abstract

Gestational diabetes mellitus (GDM) leads to increased lifelong cardiometabolic risk in both mothers and their offspring. The identification of effective strategies to contain the future risk of type 2 diabetes (T2D) and cardiovascular disease (CVD) is of utmost importance to reduce the burden of the disease. Breastfeeding (BF) is effective in reducing short- and long-term child morbidity. In recent years, BF has emerged as a candidate low-cost intervention to prevent future cardiometabolic complications both in mothers and infants exposed to GDM. The aim of this review is to provide an overview of the evidence about the possible metabolic benefits of BF for both mothers with a history of GDM and their offspring. Increasing evidence supports the positive effects of exclusive BF over formula feeding (FF) or mixed feeding on glucose homeostasis and the lipid profile in women with previous GDM in the early postpartum period. Studies with a longer observation suggest clear benefits of intensive and longer BF on the risk of diabetes and prediabetes in mothers after adjustment for confounders. In regards to infants, in most studies, the intensity and duration of BF are positively associated with slower infant growth curves compared with FF, indicating that the positive effect of BF on growth trends might contrast the increased risk of obesity and metabolic diseases observed in infants exposed to GDM. Considering these findings, a global effort should be made to support BF practice to possibly reduce cardiometabolic morbidity after GDM.

## 1. Introduction

Gestational diabetes mellitus (GDM) is a common pregnancy-related condition for which mothers are screened at 24 to 28 weeks gestational age [1]. In the last decades, the growing widespread of overweight and obesity, together with the delayed age of reproduction, have likely been the main drivers of the global rise in GDM prevalence [2].

It is well documented that GDM markedly increases the risk of maternal and offspring complications, which might develop soon after delivery or even later in life. The recurrence rate of GDM is elevated, ranging approximately between 30 and 80% [3,4,5]. In parallel, a history of GDM confers to mothers up to a 10-fold increased risk for type 2 diabetes (T2D), compared with healthy pregnancy [6]. Based on current evidence, the occurrence of postpartum glucose alterations after GDM is linked to a range of predictors, involving both maternal risk factors, including advanced age, obesity, family history of T2D, non-Caucasian ethnicity, and pregnancy-specific factors, such as high levels of glycemia at OGTT, increased HbA1c, insulin use, multiparity, hypertensive disease, and preterm delivery [7]. In addition, a history of GDM boosts by 45% the risk of overall cardiovascular and cerebrovascular diseases [8] and increases the incidence of kidney diseases in later life [9].

GDM increases the risk for offspring adverse short-term outcomes, including impaired fetal growth, birth trauma, hypoglycemia, hypocalcemia secondary to hypomagnesemia, heart malformations, respiratory distress syndrome, and feeding problems [10]. Besides the well-documented delivery complications of GDM, namely preterm birth and increased risk for cesarean delivery, mounting evidence advocates an increased risk of long-term offspring adverse outcomes, likely due to the detrimental effect of exposure to high glucose in the intrauterine environment. Specifically, offspring of mothers with GDM have increased adiposity and impaired glucose metabolism in childhood and adolescence [11,12,13,14]. In light of the link between the history of GDM and maternal and offspring adverse outcomes in the short and the long term, the identification of modifiable risk factors or habits is of utmost importance to develop appropriate management programs and effective prevention strategies.

It has been observed that early programs, introduced within three years after pregnancy, based on lifestyle interventions targeting diet and/or physical activity can largely contain the risk of diabetes in subsequent years [15].

Although data are still evolving, the breastfeeding (BF) habit has been identified as a candidate for effective low-cost intervention to prevent future cardiometabolic complications both in mothers and infants exposed to GDM.

Breastmilk is a highly complex fluid with rather unique adaptation capacities. It is under constant evolution pressure and its highly versatile composition can easily meet the nutritional and non-nutritional circumstantial needs of the offspring. Aside from providing adequate nutritional supply, human milk is enriched with key antimicrobial and immunomodulatory factors, assuring protection against infant infections [16]. BF has well-established health benefits for infants, protecting against several short- and long-term illnesses. Specifically, a protective effect in the child has been demonstrated against respiratory and gastrointestinal diseases, urinary tract infections, allergic rhinitis, asthma, atopic dermatitis, and food allergies [17,18]. Interestingly, there is also consistent evidence of an association between BF and a reduced risk for autoimmune diseases in infants, including type 1 diabetes (T1D), by around 20%, with a more marked effect observed for a BF duration of at least four months [19]. This result is of utmost interest since the incidence of T1D was found to be four-fold higher in offspring born to mothers with GDM [20].

BF has recognized additional benefits in mothers, including reduced postpartum bleeding, infections, mood disorders, sleep disturbance, and ovarian and breast cancer incidence [17,18,21].

Exclusive BF for the first six months is therefore recommended by the American Academy of Pediatrics, as well as its maintenance after solid food introduction, possibly for 2 years or beyond [22]. Relevant cardiometabolic benefits for both the mother and the infant seem to be linked to long-lasting BF. According to data from the World Health Organization, the global prevalence of exclusive BF during the initial six months postpartum exhibited a 10 percentage point increase over the past decade, attaining 48% in 2023 [23]. However, a systematic review has reported reduced practice of exclusive or predominant BF at hospital discharge following parturition in women with GDM [24]. Furthermore, the duration of exclusive or predominant BF was observed to be inferior in mothers with previous GDM in comparison with mothers without GDM [24]. Multiple factors may contribute to suboptimal BF outcomes. For instance, obese postpartum women may experience diminished milk production during the initial lactation phase [25]. Additionally, formula feeding (FF) has been correlated with cesarean delivery and preterm birth, both of which are recognized as frequent delivery complications of GDM [24,26].

The aim of this manuscript is to summarize what is currently known about the possible metabolic benefits connected with BF both for mothers with a history of GDM and for their offspring, giving an overview of the potential pathogenic mechanisms behind this association. In addition, a current picture of how this behavior is widespread among women with previous GDM is described.

## 2. BF and Glycolipid Profile in Mothers with History of GDM

It is well established that exposure to GDM has significant health implications for mothers in later life, both soon after delivery and in future years. In particular, previous GDM is a recognized risk factor for developing T2D [27,28]. Overall, in mothers exposed to GDM, the risk for future T2D increases by 4- to 10-fold in later years [6]. Given the global rise in the prevalence of GDM, the concern about a parallel increase in T2D occurrence urges the adoption of cost-effective strategies that might significantly limit the burden of GDM. Addressing these concerns, observational evidence has suggested possible beneficial actions of BF on glycolipid homeostasis in mothers with a history of GDM, mostly reporting even a positive correlation between BF duration and the magnitude of metabolic advantages, both in the short and the long term.

### 2.1. Early Effects of BF on Metabolic Health of Mothers with History of GDM

After delivery, insulin sensitivity generally restores to the pre-pregnancy state, in parallel with the discharge of the fetal placental unit, accompanied by the rapid drop in placental lactogen levels. However, impaired β-cell function can persist, and postpartum glucose intolerance might develop up to 15 years after GDM [29]. Current recommendations suggest testing women with previous GDM 4–12 weeks after delivery to rule out persistent glucose alterations. Although the proportion of women that adhere to postpartum screening is estimated to be just around 40–50% [30,31], based on the reported data, around 1.9% of women with previous GDM have T2D at postpartum screening, while the prevalence of glucose regulation impairment, defined as impaired fasting glucose (IFG) and/or HbA1c 5.7–6.5% and/or impaired glucose tolerance (IGT), ranges from 7.9% to 18% [30,32,33,34,35]. Given the high proportion of women with residual glucose alterations after GDM, the early postpartum period seems to be a critical window for interventions aimed at preventing T2D. Besides the proven efficacy of lifestyle interventions and metformin therapy in delaying progression to T2D, evidence has highlighted a consistent link between BF and glucose and lipid profile improvement in women with previous GDM in the early postpartum period (Table 1).

Relevant information ensued from the results of the “Study of Women, Infant Feeding, and Type 2 Diabetes” (SWIFT), a prospective cohort study that recruited more than 1000 women with GDM at 20–37 weeks of gestation [36]. A baseline evaluation was performed at 6–9 weeks postpartum, and the follow-up period ended 2 years after delivery. At baseline, patients underwent a 75 g oral glucose tolerance test (OGTT), and fasting plasma glucose (FPG), fasting insulin (FI), 2 h (2 h) post-load insulin, and a homeostasis model assessment of insulin resistance index (HOMA-IR) were correlated to BF intensity in a subgroup of 522 women reporting information about lactation habits. In the linear regression model, exclusive BF and predominantly BF arms showed a significant decrease in FPG by around −4.0 mg/dL and −5.0 mg/dL, respectively, as well as a reduction in FI, 2 h post-load insulin, compared with exclusively or mostly FF after adjustment for several confounders, including parity, race, age, BMI, weeks postpartum, and education. Conversely, the effect of lactation intensity on 2 h post-load glucose levels was not significant. The high intensity of BF (exclusive and mostly BF) was also associated with a lower occurrence of prediabetes and diabetes at OGTT [36]. The benefits of intensive lactation on glucose homeostasis were confirmed in another analysis of the SWIFT, including a larger cohort of patients with previous GDM (*n* = 1007), without persistent diabetes at baseline evaluation (6–9 weeks postpartum) [37]. The authors reported that exclusive BF and mostly BF groups displayed reduced values of FPG, FI, 2 h post-load glucose, and 2 h post-load insulin, as well as higher insulin sensitivity and insulin secretion indices. Of note, a lower proportion of individuals in high-intensity groups were obese and had IGT [37].

In a sub-analysis of two large prospective studies including 1008 women with previous GDM, exclusive and mixed BF groups displayed a lower rate of glucose alterations in an OGTT (T2D or prediabetes) performed in early postpartum than the group that did not breastfeed. Nevertheless, the effect was largely attenuated when adjusting for covariates, including pre-pregnancy BMI, race, education, and income, becoming no longer significant for the group with mixed BF compared with the no BF group [38]. Of note, women in the BF group exhibited a better metabolic profile compared with the other two groups, hallmarked by a lower BMI, weight retention, triglycerides, and HOMA-IR.

Similarly, a recent prospective study that included 171 women participating in the “MySweetheart trial” evaluated the relationship between BF and metabolic outcomes up to 1 year after delivery. An inverse association of BF duration with weight, weight retention, visceral adipose tissue, insulin resistance indices, and C-reactive protein after adjustment for pre-pregnancy BMI, education, and treatment for GDM was found. Conversely, a positive association between insulin secretion indices and BF length was reported [39]. When stratifying for BF duration, the group who breastfed for more than 6 months displayed a better metabolic profile compared with the group with a BF duration of less than 6 months, although significance was not confirmed adjusting for pre-pregnancy BMI, education degree, and glucose-lowering therapy during pregnancy [39].

The benefit of BF on early postpartum FPG (6–10 weeks) was confirmed in another study involving 243 women, 159 with a history of GDM, and 84 control subjects [40]. Interestingly, exclusive BF was linked to a decrease in FPG by 3.4 mg/dL compared with women who did not exclusively BF, regardless of the presence of a positive history of GDM. When restricting the analysis to women with previous GDM, the group reporting BF had lower FPG compared with women who did not exclusively BF [40].

Accordingly, a sub-analysis of the “Atlantic Diabetes in Pregnancy (Atlantic DIP) study”, including 300 women with previous GDM and 220 control subjects who underwent diabetes screening 12 weeks after delivery with 75-g OGTT, pointed out that OGTT alterations after delivery were significantly less frequent in the BF group compared with the FF group. Of note, in the multivariate model, adjusting for BMI, age, ethnicity, insulin therapy during pregnancy, family history of T2D, and metabolic syndrome, BF reduced by 58% the likelihood of glycemic alterations at OGTT [33].

In a Japanese multicenter observational prospective study, 222 women with previous GDM were tested with an OGTT at 6–12 weeks after delivery. The high-intensity BF group, including exclusive BF and mostly BF (minimal FF and more than 80% of the volume by BF), had significantly lower FPG and fasting insulin at OGTT, as well as lower HOMA-IR than women in the non-intensive BF group. In a multivariate model adjusted for confounders, including age, pre-pregnancy BMI, family history of T2D, FPG at the diagnosis, insulin therapy during pregnancy, and delivery factors, high-intensity BF was associated with lower HOMA-IR values, while non-intensive BF was an independent predictor of high insulin resistance (HOMA-IR ≥ 75th percentile). However, when stratifying for pre-pregnancy BMI, intensive BF had a significant effect on insulin sensitivity only in obese women [41].

In a retrospective Italian study, 97 women with a history of GDM (81 BF and 16 no BF) were screened for diabetes with an OGTT at 12–16 weeks after delivery [42]. The BF group had significantly lower values of FPG, 2 h post-load glucose, triglycerides, and HOMA-IR, as well as lower prevalence of IFG and IGT.

In a recent prospective study, the rates of glucose alterations, according to BF behavior, were evaluated in 130 women with previous GDM, 6 months after delivery. Maintaining BF at six months accounted for a reduction in the rate of prediabetes. Noteworthy, only in the exclusive BF group, protection against prediabetes was significant, not in the partial BF group [43].

Overall, studies that have primarily focused on the effects of BF on glucose metabolism in the early postpartum period in women with a history of GDM have indicated a clear beneficial influence of BF intensity on FPG, which was around 3–5 mg/dL lower in exclusive or mostly BF groups over the FF group. In addition, women who breastfed after the index pregnancy were shown to have lower post-load glucose values. These findings are in line with the consistent evidence of a decreased amount of visceral adipose fat in women who breastfed, which leads to reduced insulin resistance and glucose homeostasis improvement. Indeed, most studies have observed a significant association between BF and a decrease in insulin resistance indices. Importantly, besides the improvement in insulin sensitivity, a sustained amelioration of insulin secretion indices in BF groups has also been reported, suggesting that even beta-cell function might benefit from intensive BF. The vast majority of studies have therefore shown a clear protective role of exclusive BF over FF or mixed feeding on glucose alterations at a postpartum OGTT, consistently reporting a reduced incidence of prediabetes and diabetes. Interestingly, although attenuated, a beneficial effect of mixed feeding has emerged over no BF, suggesting that, when feasible, BF should be always encouraged to achieve better outcomes in the early postpartum period.

In regards to lipid changes, an analysis that included women enrolled in the SWIFT, without diabetes at baseline evaluation performed 6–9 weeks postpartum, revealed a significant graded association between increasing lactation intensity (from mixed to exclusive BF compared with exclusive or mostly FF) and lower triglycerides, higher high-density lipoprotein cholesterol (HDL-cholesterol) and lower leptin levels, after adjustment for ethnicity, education, weeks postpartum, pre-pregnancy BMI, and minutes BF during fasting period [37].

In a recent study, with a nested case-control design, involving a sample of around 300 women enrolled in the SWIFT with a history of GDM, with normal glucose metabolism at basal evaluation (6–9 weeks after delivery), a prospective evaluation for diabetes occurrence at 2 years and at 8 years was performed [44]. Targeted lipidomics and metabolomics were performed in fasting samples both at baseline and after 2 years in order to identify metabolites that were predictive of T2D risk in relation to BF intensity (intensive BF compared with intensive FF or mixed feeding). Lactation intensity was associated with significant changes in maternal lipid profiles at baseline. Specifically, triacylglycerols and diacylglycerols, used for milk production, were negatively associated with intensive BF, while an increase in phospholipids and sphingolipids during lactation was found. Phospholipids and sphingolipids play a key role in cell signaling, and their reduction is linked to the development of insulin resistance, likely due to impaired insulin receptor signaling [45]. However, these lipid profile changes were not observed 2 years after delivery, reasonably in line with the end of BF practice. Furthermore, a favorable lipid profile was not observed in the group that developed T2D during the follow-up [44].

Conversely, Shub et al. did not observe significant differences in HDL-c, triglycerides, and LDL-c between the exclusive BF group and the not exclusive BF group after adjustment for covariates, including previous GDM in the early postpartum period (6–10 weeks) [40].

Although the number of studies exploring lipid changes related to BF in the early postpartum period is still limited, an improvement in triglyceride levels seems to be consistently favored by intensive BF. This effect is largely attributable to the augmented uptake of triacylglycerols and diacylglycerols by the mammary gland for milk production during lactation. Conversely, the effect of BF on HDL-cholesterol and LDL-cholesterol needs further investigation, given the inconsistency of the available results.

**Table 1:** Studies evaluating the relationship between lactation and metabolic changes in mothers with previous GDM in the early postpartum period.

Study	Study Design	Population	Groups	Postpartum Evaluation	Lactation Classification	Main Findings
Gunderson et al., 2012 [36]	Prospective	GDM *n* = 522	Exclusive BF (*n* = 211) Mostly BF (*n* = 99) Mixed or inconsistent (*n* = 77) Exclusive or mostly FF (*n* = 135)	6–9 wks	Exclusive BF: no formula; Mostly BF: ≤6 oz formula/24 h; Mixed or inconsistent feeding of breast milk and formula: 7–17 oz/24 h or change in feeding status to increase formula Exclusive or mostly FF: >17 oz formula/24 h	Exclusive BF and mostly BF vs. exclusive or mostly FF: lower FPG, FI, and 2 h insulin; lower prevalence of diabetes or prediabetes
Shub et al., 2019 [40]	Prospective	GDM *n* = 159 NGT *n* = 84	Exclusive BF (*n* = 106) Not exclusive BF (*n* = 53)	6–10 wks	BF status categorized as exclusively BF or not exclusively BF, including women who were feeding both formula and BF	GDM group Exclusive BF vs. not exclusive BF: lower FPG, no difference in lipids
O’Reilly et al., 2011 [33]	Prospective	GDM *n* = 300 NGT *n* = 220	BF (*n* = 319) FF (*n* = 20)	12 wks	All the following criteria required for lactation definition: ongoing feeding (at least 4 times per day; meeting maternal expectations; duration > 8 wks; infant reaching developmental milestones, in particular, gaining weight; infant receiving scheduled immunizations	BF vs. FF: lower prevalence of persistent OGTT alterations
Yasuhi et al., 2019 [41]	Prospective	GDM *n* = 222	High-intensity BF (*n* = 166) Non-high intensity BF (*n* = 56)	6–12 wks	High-intensity BF: “BF alone”, “mostly BF with a minimal additional formula”, and “≥80% of the volume by BF; <20% by formula” Non-high intensity BF: “60–70% by BF; 30–40% by formula”, “≤50% by BF; >50% by formula”, and “by formula alone”	High-intensity BF vs. non-high-intensity BF: lower FPG and FI at OGTT; In obese patients: higher HOMA-IR in non-high intensity BF vs. high-intensity BF
Corrado et al., 2019 [42]	Retrospective	GDM *n* = 97	BF (*n* = 81) No BF (*n* = 16)	3–4 mo	NA	BF vs. no BF: lower FPG, 2 h glucose, TG, HOMA-IR; lower prevalence of IFG and IGT
Gunderson et al., 2014 [37]	Prospective	GDM *n* = 1007	Exclusive BF (*n* = 437) Mostly BF (*n* = 183) Mixed or inconsistent breast milk and formula (*n* = 128) Mostly or exclusively FF (*n* = 259)	6–9 wks	Exclusive BF: 0 ounces of formula; mostly BF: ≤6 ounces of formula/24 h; mixed (breast milk and formula >6 to ≤17 ounces/24 h) or inconsistent feeding method; mostly FF > 17 ounces/24 h; exclusive FF	Exclusive BF and mostly BF groups vs. mostly or exclusive FF groups: lower fasting glucose, FI, 2 h glucose 2 h insulin; higher insulin sensitivity index; lower insulin resistance and insulin secretion indices; less likely to be glucose intolerant ↑ lactation intensity: ↓ TG, ↑ HDL-c, ↓ leptin
Vanlaer et al., 2024 [38]	Prospective	GDM *n* = 1008	Exclusive BF (*n* = 567) Mixed BF/FF (*n* = 102) Exclusive FF (*n* = 339)	12 wks	Exclusive BF (<45 mL FF/day); mixed BF/FF; exclusive FF (≥150 mL FF/day)	Exclusive and mixed BF vs. no BF: ↓ rate of T2D, prediabetes. The effect was no longer significant for the mixed BF group when adjusting for pre-pregnancy BMI, race, education, and income.
Hebeisen et al., 2024 [39]	Prospective	GDM *n* = 171	BF < 6 mo (*n* = 69) BF ≥ 6 mo (*n* = 102)	1 yr	BF < 6 mo; BF ≥ 6 mo	Inverse association of BF duration with weight, weight retention, visceral adipose tissue, insulin resistance indices, and C-reactive protein after adjustment for pre-pregnancy BMI, education, and therapy during pregnancy
Zhang et al., 2021 [44]	Prospective	GDM *n* = 350	Intensive BF (*n* = 216) Intensive FF (*n* = 134)	6–9 wks	BF intensity and duration ratio: the number of breast milk feeds/24 divided by the total number of all liquid feeds/24 h during the past 7 days to yield a score from 0 to 1. 1 = exclusive BF, 0 = exclusive FF, fractional scores = levels of lactation intensity	Intensive BF: ↓ TG and DAG; ↑ phospholipids and sphingolipids
Suthasmalee et al., 2024 [43]	Prospective	GDM *n* = 130	BF maintained at 6 mo (exclusive BF *n* = 49 or partial BF *n* = 24) BF < 6 mo *n* = 57	6 mo	BF maintained at 6 mo (exclusive BF or partial BF) BF < 6 mo	Maintaining BF at 6 mo vs. BF < 6 mo ↓ rate of prediabetes. The protective effect of BF against prediabetes was significant only in the EBF group, not in the partial BF group.

GDM: gestational diabetes mellitus; NGT: normal glucose tolerance BF: breastfeeding; FF: formula feeding; BMI: body mass index; FPG: fasting plasma glucose; FI: fasting insulin; OGTT: oral glucose tolerance test; IFG impaired fasting glucose; IGT impaired glucose tolerance; TG: triglycerides; HOMA-IR: homeostasis model assessment-insulin resistance; HDL-c: high-density lipoprotein-cholesterol; DAG: diacylglycerols; wks: weeks; mo: months; yr: year; ↑ = increase; ↓ = decrease.

### 2.2. BF and GDM Recurrence

GDM recurrence is highly frequent in women with previous GDM and the possible benefits of lactation on recurrence rate have been poorly investigated so far. In a cohort study, recurrence of GDM has been retrospectively evaluated in a population of 229 women with GDM diagnosis in the index pregnancy. Among them, 139 reported exclusive BF for at least one month after delivery, while 90 women introduced bottle feeding earlier. The rate of GDM recurrence in the subsequent pregnancy was reduced by 44% in the exclusive BF group after adjustment for parity [46].

In another study that enrolled 220 women with a history of GDM undergoing GDM screening in a subsequent pregnancy, intensive BF was associated with lower 2 h post-load glucose and with a 22% reduced risk of GDM recurrence with respect to the low-intensity BF group after adjustment for age and BMI. In addition, women reporting a BF duration longer than six months displayed lower mean 1 h glucose at OGTT and a 19% reduced risk of GDM recurrence compared to a BF duration lower than 6 months, adjusting for BMI, maternal age at second pregnancy, GDM therapy in first pregnancy, and ethnicity [47]. The same authors, in a larger sample of women with high GDM risk (including patients with previous GDM), observed a significant relationship between intensive BF in the first three months after delivery and an 18.3% reduced risk of FPG alterations at OGTT in a subsequent pregnancy compared to low-intensity BF [48].

Although data are still evolving, these results are suggestive of a positive influence of intensive and prolonged BF after delivery on GDM recurrence in future pregnancies.

### 2.3. Long-Standing Effects of BF on Metabolic Health of Mothers with History of GDM

Other studies have investigated the effect of BF on maternal metabolic profile over a longer period of time after delivery (Table 2). In particular, several studies have evaluated long-term T2D risk in mothers with previous GDM after the index pregnancy.

The follow-up of the SWIFT reported an incidence of diabetes of 11% two years after GDM diagnosis in women without diabetes at baseline evaluation (6–9 weeks postpartum) [49]. Of note, diabetes occurrence was less frequent in women reporting higher lactation intensity, adjusting for age and other maternal and newborn-related confounders. Moreover, a longer BF duration is independently associated with a reduction in adjusted diabetes incidence, relative to the group reporting short BF duration (0–2 months) [49].

O’Shea et al. evaluated the incidence of IFG, IGT, and T2D in 79 women with GDM history four years after delivery [50]. BF resulted to be a protective factor, reducing by 84% the risk for OGTT impairment, and importantly, the risk was not attenuated when adjusting for confounders, such as age, ethnicity, relatives with T2D, weight gain, or occupation.

Similar results ensued from the longitudinal evaluation of an Asian cohort study, involving a subset of women with GDM diagnosis in the index pregnancy (*n* = 116) enrolled in the “Growing Up in Singapore Towards healthy Outcomes (GUSTO) study” [51]. The women were followed up, and a 75 g OGTT was performed four to seven years after delivery with the aim of evaluating the effect of BF duration on T2D and prediabetes risk. The group of women who reported a BF duration longer than 6 months had a 50% reduced incidence of OGTT impairment (T2D or prediabetes) compared with women not performing BF or with a lactation duration <1 month. Of note, this association remained significant after adjustment for multiple covariates, such as age at delivery, parity, a family history of T2D, pre-pregnancy BMI, and smoking habits [51].

In another study involving 1649 women with a history of GDM and 3243 without a history of GDM, the main predictors of T2D incidence over 10 years of observation were identified [52]. A higher frequency of BF was associated with a significant reduction in T2D incidence (around 28%) in a multivariate model including age, parity, history of GDM, regular physical activity, family history of T2D, and history of stillbirth or abortion.

Gunderson et al. evaluated T2D risk in women enrolled in a community-based cohort study, the 30-year “Coronary Artery Risk Development Study in Young Adults (CARDIA)” [53]. The analysis involved 1238 women without pre-pregnancy diabetes and 155 with GDM diagnosis in the index pregnancy who reported information about lactation duration. Lactation duration groups were defined as no BF, more than 0 to 6 months, more than 6 months to less than 12 months, and 12 months or more. During the 30 years of observation, diabetes incidence was higher for women with GDM than women without GDM. Both among women with and without GDM, longer BF was associated with reduced diabetes incidence. Of note, the subgroup reporting a lactation duration longer than 12 months had the lowest incidence of diabetes. In multivariate models, a graded link between longer lactation duration categories and lower diabetes incidence emerged, with confounders, such as ethnicity, pre-pregnancy BMI, waist circumference, FPG, HOMA-IR, age, parity, history of GDM, and T2D having a marginal effect on the model’s significance. Overall, the group reporting a lactation duration longer than 6 months had a 25–47% reduction in diabetes risk compared with the group who breastfed less than six months [53].

In a prospective German study involving 304 women, T2D incidence was evaluated with a 75 g OGTT periodically performed over 20 years of observation. The incidence of diabetes was 63.6% in women with a history of GDM. Lactation duration was inversely associated with diabetes risk, and women performing BF for more than 3 months had a 45% lower risk of diabetes compared with women reporting no BF or BF for less than 3 months. However, the effects of potential confounders have not been evaluated in a multivariate model [54].

The results of the “Diabetes & Women’s Health (DWH) Study”, which enrolled 577 women 9–16 years after a pregnancy complicated by GDM, are in contrast with the previously reported findings that reported an association of longer lactation duration with a better maternal cardiometabolic profile later in life. Specifically, herein, a longer lactation duration was not predictive of a parallel reduction in the risk of T2D, prediabetes, and obesity after adjustment for pre-pregnancy BMI, parity, and lifestyle factors [55].

The “Nurses’ Health Study (NHS)” and the “Nurses’ Health Study II (NHS II)” prospectively evaluated the association of total lifetime duration of lactation with incident T2D. In both cohorts, duration of lactation was inversely associated with T2D incidence. In particular, T2D risk reduction varied between 4 and 16% for each additional year of lactation, adjusting for parity, BMI at 18 years, dietary habits, physical activity, family history of diabetes, smoking status, and birth weight of participants. However, when stratifying by a diagnosis of GDM, the duration of BF had no significant effect on T2D incidence in women with a history of GDM [56]. Contrarily, in an updated retrospective analysis involving 4372 women with previous GDM participating in the NHS II, comprising a follow-up of 25 years, namely 20 years longer than the previous evaluation, both a longer lifetime lactation duration and longer exclusive lifetime duration showed a lower risk of T2D after adjustment for relevant confounders, including after adjustment for age, ethnicity, family history of diabetes, parity, age at first birth, and lifestyle factors. In addition, an inverse long-term association between BF duration and A1c, as well as with insulin and C-peptide, was observed in women not developing T2D [57].

A recent meta-analysis evaluated the association of BF with T2D/prediabetes risk or metabolic parameter changes in women with previous GDM over a follow-up period that ranged between 6 weeks and 24 years after delivery. Longer BF was related to a 21% reduced risk of T2D in the follow-up period, as well as to a decrease of 34% in the risk of IFG/IGT, independently of BF intensity. Of note, the beneficial effect of BF on T2D risk did not emerge in the early postpartum period but became significant when the follow-up length of the studies was longer than one year. A significant effect of high BF intensity was also observed, compared with no BF, in a follow-up period of 1–5 years postpartum. In regard to metabolic parameters, longer lactation was associated with reduced FPG, HOMA-IR, and triglycerides [58].

Overall, although some inconsistencies between studies have emerged, likely due to not homogeneous patient populations (ethnicity and diabetes prevalence in the population enrolled) and the study methods (diagnostic criteria for GDM, self-reported T2D diagnosis, follow-up length, lactation classification, and assessment of BF), a growing body of evidence supports clear benefits of BF on metabolic profile and prediabetes/T2D risk in mothers with previous GDM even in the long-term, with most studies emphasizing a quite attenuated effect of mixed BF in comparison with exclusive BF. In addition, consistent evidence supports a graded inverse association of BF duration with glucose impairment incidence, even after adjustment for confounders. It should be highlighted that the evidence of benefit largely ensues from prospective observational studies with potentially unknown confounders, given that randomized controlled trials assigning women to either BF or not BF would raise relevant ethical issues.

**Table 2:** Studies evaluating the relationship between lactation and metabolic changes in mothers with previous GDM in the long term.

Study	Study Design	Population	Groups by BF Habits	Follow-Up	Lactation Classification	Main Findings
Gunderson et al., 2015 [49]	Prospective	*N* = 1010	Exclusive BF (*n* = 205) Mostly BF (*n* = 387) Mostly FF, Mixed/ inconsistent (*n* = 214) Exclusive FF (*n* = 153)	2 yrs	Exclusive BF (no FF), mostly BF (>0 to 6 oz of formula per 24 h), mostly FF (>17 oz per 24 h), and mixed (7 to 17 oz of formula per 24 h) or inconsistent lactation pattern, and exclusive FF (formula only; no BF or BF < 3 weeks)	Inverse association for lactation intensity at baseline and duration with incident T2D
O’Shea et al., 2023 [50]	Retrospective	*N* = 74	BF (*n* = 50) No BF (*n* = 24)	4 yrs	BF status categorized as any BF yes or no	BF and BF duration associated with lower likelihood of abnormal glucose tolerance, adjusting for age, ethnicity, relatives with T2D, weight gain, and occupation
Hewage et al., 2021 [51]	Retrospective	*N* = 116	No BF or <1 (*n* = 21) >1 to <6 (*n* = 50) >6 mo (*n* = 45)	4–7 yrs	No BF or <1, >1 to <6, and >6 mo	BF duration > 6 mo 50% reduced incidence of OGTT alteration compared with women who did not breastfeed or with lactation duration < 1 mo
Feleke et al., 2020 [52]	Prospective	*N* = 1649	NS	10 yrs	BF status categorized as any BF yes or no	Frequency of BF inversely associated with T2D incidence, adjusting for age, parity, history of GDM, regular physical activity, family history of T2D, and history of stillbirth or abortion
Gunderson et al., 2018 [53]	Prospective	*N* = 155	NS	30 yrs	BF duration: none; <6 weeks; 6–11 weeks; 3–6 mo; or 6 mo or more	Graded inverse association between longer lactation duration and diabetes incidence, adjusting for ethnicity, pre-pregnancy BMI, waist circumference, FPG, HOMA-IR, age, parity, and history of GDM and T2D
Ziegler et al., 2012 [54]	Prospective	*N* = 304 (*N* = 264 with BF information)	No BF (*n* = 63) BF < 3 mo (*n* = 109) BF > 3 mo (*n* = 92)	20 yrs	BF duration: no BF; BF ≤ 3 mo; BF > 3 mo	Duration of lactation inversely associated with postpartum diabetes risk. BF > 3 mo had 45% lower risk of diabetes compared with no BF or BF < 3 mo
Wander et al., 2022 [55]	Prospective	*N* = 577 (*N* = 532 with BF information)	No BF (*n* = 57) <6 mo (*n* = 101) 6–12 mo (*n* = 171) 12–24 mo (*n* = 143) ≥24 mo (*n* = 60)	9–16 yrs	No BF; <6; 6–12; 12–24; ≥24 mo	Longer lactation duration not predictive of reduced risk of T2D, prediabetes, and obesity after adjustment for pre-pregnancy BMI, parity, and lifestyle factors
Stuebe et al., 2005 [56]	Retrospective	*N* = 266	No BF (*n* = 265) >0 to 3 mo (*n* = 197) >3 to 6 mo (*n* = 114) >6 to 11 mo (*n* = 185) >11 to 23 mo (*n* = 224) >23 mo (*n* = 147)	10 yrs	No BF >0 to 3 mo >3 to 6 mo >6 to 11 mo >11 to 23 mo >23 mo	Duration of lactation inversely associated with T2D incidence but no significant effect when stratifying by GDM history
Ley et al., 2020 [57]	Prospective	*N* = 4372	No BF (*n* = 766) >0 to 6 mo (*n* = 770) >6 to 12 mo (*n* = 871) >12 to 24 mo (*n* = 1082) >24 mo (*n* = 833)	25 yrs	No BF >0 to 6 mo >6 to 12 mo >12 to 24 mo >24 mo	Longer lifetime lactation duration and longer exclusive lifetime duration associated with lower risk of T2D after adjustment for age, ethnicity, family history of diabetes, parity, age at first birth, and lifestyle factors

GDM: gestational diabetes mellitus; BF: breastfeeding; FF: formula feeding; OGTT: oral glucose tolerance test; T2D: type 2 diabetes; FPG: fasting plasma glucose; HOMA-IR: homeostasis model assessment of insulin resistance index; NS: not specified; mo: months; yrs: years.

## 3. BF and Progression to T2D in Mothers: The Mechanisms Behind the Possible Beneficial Effects

**Figure 1:**
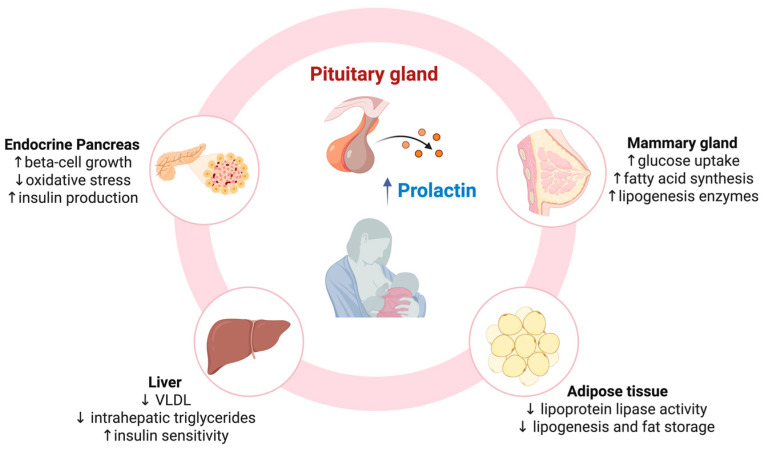
Mechanisms supporting the metabolic effects of BF in mothers. VLDL: very low density lipoprotein; ↑ = increase; ↓ = decrease.

Current epidemiological evidence strongly suggests that BF plays a protective role against the development of T2D in mothers, particularly in those with previous GDM. The underlying mechanisms that explain this effect are largely unknown, although a multitude of players are supposed to be involved, including the mammary gland, the pituitary gland, the white adipose tissue, the liver, and the endocrine pancreas (Figure 1). These organs undergo significant metabolic adaptations during lactation, which not only support milk production but also seem to have a long-term impact on maternal metabolic health. An intricate network involving a multitude of players supports the profound adaptive metabolic response to lactation (Figure 1). Hormone modifications, which begin during pregnancy and evolve after delivery, seem to be the main driver of the metabolic changes induced by BF. A central role in the complex interplay that leads to glucose homeostasis modifications during lactation is played by the pituitary hormone prolactin, which critically mediates the crosstalk between the pituitary gland, the mammary gland and insulin target organs, such as the adipose tissue, the liver, and the skeletal muscle. Even the endocrine pancreas is invested by profound dynamic modifications, likely orchestrated by hormonal changes, which conduct secretion function improvement during lactation. Herein, the relative contributions of the main players in this complex network are summarized.

### 3.1. Role of the Mammary Gland in Glucose Homeostasis During Lactation

The mammary gland plays a pivotal role in the metabolic modifications observed in BF women. It enhances the utilization of glucose and lipids, thereby facilitating effective milk synthesis [59]. In parallel, during lactation, the increased delivery of glucose to the mammary gland promotes circulating glucose and insulin clearance, thus reducing blood glucose levels [60,61]. The mammary gland expresses insulin receptors [62]. Insulin within the mammary gland activates various secondary messengers that promote glucose uptake via glucose transporter 1 (GLUT1) and the synthesis of non-esterified fatty acids. Activation of the prolactin receptor triggers signaling pathways that upregulate the expression of insulin receptor substrate 1 (IRS-1) and overexpress enzymes that augment lipogenesis [59]. Besides this, glucose uptake is largely sustained by insulin-independent pathways [63]. In a sub-analysis of the SWIFT, excluding women reporting exclusive FF, the effect of BF while performing the 75 g OGTT on several metabolic parameters was evaluated. BF during the test led to lower maternal 2 h post-load glucose and insulin levels compared without BF, adjusting for covariates, including age, ethnicity, parity, GDM severity, the amount of formula per 24 h, frequency of feedings per day, and the duration of fasting, while basal insulin and glycemia, as well as insulin resistance indices, were not significantly influenced [64]. The reduced levels of 2 h glucose might be related to the insulin-independent glucose uptake [65]. However, in a recent randomized clinical trial, 2 h post-load glucose was not significantly influenced by BF during OGTT in women with previous GDM early after delivery [66]. Further investigations are therefore needed to clarify whether glucose sensitivity is modified during BF due to glucose uptake by the mammary gland.

The metabolic modifications in the mammary gland seem to be crucially driven by the activation of the mammalian target of the rapamycin (mTOR) pathway [59]. This protein kinase is ubiquitously expressed throughout the body and is essential to several organs implicated in the pathogenesis of T2D. In the hypothalamus the signal peptide Nesfatin-1 seems to influence mTOR pathway activation in the liver, regulating insulin signaling and hepatic glucose metabolism [55]. In skeletal muscle and adipose tissue, it modulates insulin sensitivity and is essential for maintaining muscle function. In the pancreas, mTOR plays a role in the proliferation of β-cells [9]. In addition, in the context of BF, the mTOR pathway leads to the activation of different enzymes (e.g., fatty acid synthase gene (FasN), Hepatic ATP-citrate lyase (ACLY), malic enzyme (ME), and stearoyl-CoA desaturase 2 and 3 (SCD-2/-3)), which are involved in increasing lipogenesis, necessary for milk synthesis [59].

### 3.2. Prolactin-Mediated Crosstalk Between Mammary Gland and Adipose Tissue in Metabolic Homeostasis

Prolactin is crucially involved in the regulation of lactation. It has a trophic effect on the mammary alveoli, enhances the expression of genes encoding milk constituents, and regulates postpartum milk production stimulated by infant suckling [67]. Interestingly, recent literature indicates that prolactin’s function extends beyond its traditional target organ. Evidence about the association of circulating prolactin levels with metabolic risk is conflicting. Chronic elevation of prolactin has been associated with impaired glucose tolerance and an increased risk of T2D [68]. Recent evidence revealed that low levels of prolactin may adversely affect glucose homeostasis, implying that maintaining prolactin levels within a physiological range is crucial for optimal glucose metabolism [68]. Interestingly, higher prolactin levels in pregnancy during the late second and early third trimester appear to be predictive of T2D development [69]. In regards to the postpartum period, an ancillary study including women participating in the SWIFT study has shown that higher circulating levels of prolactin, linked to intensive and longer BF after delivery, were linked to a lower incidence of T2D. Notably, the negative correlation between prolactin and basal glucose levels, insulin resistance indices, and fasting leptin concentration, as well as the association of prolactin with a favorable lipid profile in the subgroup of women not developing T2D, suggested that prolactin levels might be closely linked to maternal glucose and lipid changes in the early postpartum period, likely influencing future T2D risk [70]. Although the mechanisms behind this association are yet to be elucidated, preclinical evidence has revealed that prolactin plays a crucial role in glucose homeostasis, regulating both peripheral insulin sensitivity and beta-cell function [71,72,73]. Thus, the protective effect of intensive BF on T2D risk might be explained by the physiological modifications in glucose and lipid homeostasis promoted by high prolactin levels in lactating women.

### 3.3. White Adipose Tissue Adaptation During Lactation

White adipose tissue plays a crucial contributor to the development of insulin resistance, a hallmark of metabolic diseases. During lactation, adipose tissue undergoes significant metabolic adaptations to cover the amplified fuel request for milk production. One notable change is the reduction in lipoprotein lipase (LPL) activity in the adipose tissue of lactating women [59]. LPL is involved in the hydrolysis of circulating triglyceride-rich lipoproteins, facilitating the uptake of free fatty acids into adipocytes for storage [74]. The reduced activity of LPL in subcutaneous adipose tissue during lactation favors the decrease in lipogenesis and a concomitant reduction in adipose mass. This metabolic shift is crucial for reallocating energy substrates to the mammary glands, where they are utilized for milk synthesis. Given the reduction in LPL activity, an increase in circulating triglyceride levels might be hypothesized. However, epidemiological data suggest that intense and long BF is correlated to significantly reduced levels of circulating triglycerides [58]. This shows the importance of the mammary gland in utilizing glucose and triglyceride for milk production. Specifically, it uses glucose and triglycerides for the production of lactose and milk fat, respectively. Prolactin plays once more a relevant role in this metabolic pathway. Indeed, prolactin, in conjunction with growth hormone and decreased insulin levels, can inhibit LPL function [59]. Once again, the long-term effects of BF manifest in women’s later stages of life. A prospective 10-year cohort observational study, which assessed the body composition of 168 parous women, demonstrated that BF is correlated with improved body composition. With respect to non-BF women, extended BF duration (at least one year) led to lower fat mass index, reduced total fat mass, decreased tissue percentage fat, and lower visceral adipose tissue volume [75].

### 3.4. Role of the Liver in Metabolic Homeostasis During Lactation

The liver is involved in glucose metabolism. Hepatic cells modulate glucose levels by promoting glucose and insulin-mediated glycogen synthesis in the post-prandial period. On the other hand, it provides glucose by glycogenolysis and gluconeogenesis during fasting periods. Recent evidence suggests that lactation induces functional modifications in hepatic metabolism, particularly in glucose and lipid pathways. A study utilizing proton magnetic resonance spectroscopy demonstrated that lactating women exhibited significantly lower very-low-density lipoprotein triglyceride (VLDL-Tg) concentrations compared to non-lactating controls. Intriguingly, both cohorts displayed comparable modified intrahepatic triacylglycerol content (IHTG) and de novo lipogenesis rates following hyperinsulinemic–euglycemic clamp [76]. These findings indicate that lactation-induced reduction in VLDL-Tg concentrations occurs independently of IHTG and the lipogenesis rate. In addition, a direct correlation was seen between prolactin concentration and reduced IHTG in lactating women [76]. A separate cross-sectional analysis revealed a strong association between IHTG and adipose tissue insulin resistance [77]. This correlation suggests that the prolactin-mediated reduction in IHTG could potentially elucidate, at least partially, the protective mechanism conferred by BF on maternal metabolic health.

These findings underscore the complex interplay between lactation, hepatic lipid metabolism, and long-term metabolic outcomes in women.

### 3.5. Endocrine Pancreas Response to Lactation

In vitro and in vivo evidence showed that the endocrine pancreas is invested by profound changes since the beginning of BF in lactating women [78]. In detail, beta-cells are partially lost in the early days of lactation, followed by beta-cell neogenesis [78]. The exact mechanism of beta-cell mass reduction in the early phase is still unknown, but it appears to be related to apoptosis and phagocytosis. On the other hand, lactation seems to promote beta-cell proliferation and its mass expansion and reduce oxidative stress. The physiological mechanism appears to involve serotonin production in beta-cells mediated by prolactin [79], showing once again its potential protective role in progression to T2D.

## 4. Impact of BF on Metabolic Profile and Risk of T2D in Offspring of Mothers with Previous GDM

The contribution of BF on metabolic outcomes in children of mothers with prior GDM has been evaluated both early after delivery and in subsequent years. Although studies are still limited, there is growing interest in evaluating the growth trajectories of offspring during the first months and years after delivery, as a strong link seems to exist between a sharp initial weight increase and excessive adiposity in future years, independently of a history of GDM in mothers [80].

In a Danish cohort study involving *n* = 131 singleton offspring born to mothers with GDM, treated with a lifestyle intervention approach during pregnancy, information about feeding habits (fully, partly, or no BF) and offspring parameters were obtained at birth and after five weeks and five months. Both within a few weeks and five months after delivery, breastfed infants had significantly lower BMI compared with WHO standards, after adjustment for maternal pre-pregnancy BMI and smoking status [81].

The “SWIFT Offspring Study” also provided an evaluation of infant growth related to feeding patterns in the first year after in utero exposure to GDM. Although infant growth trajectories did not differ in the first 6–9 weeks across groups with different BF intensity, the intensive FF group showed a sharper increase in mean weight-for-length and weight-for-age after 6–9 weeks to 6 months and one year compared with intensive BF group, adjusting for multiple gestational, maternal and neonatal factors. Of note, a transition within 9 months to FF was also associated with growth acceleration, similar to formula-fed infants since birth [82].

The “pregnancy and neonatal diabetes outcomes in remote Australia (PANDORA) study”, a prospective longitudinal study involving *n* = 122 infants of mothers with GDM, led to similar findings. Specifically, predominant BF was associated with significantly inferior weight and BMI trajectories in infants of women with prior GDM at six months compared to mostly FF. At 14 months, a significant association between weight-for-age and mostly BF was observed in infants of women with GDM. However, statistical significance was not confirmed after adjusting for maternal characteristics, including BMI, suggesting that this variable may critically contribute to attenuating the influence of BF on infant growth outcomes [83].

An Asian prospective study reported the opposite results. In particular, BF longer than four months led to slower weight and BMI increase only in infants born to mothers without GDM over the first 12 and 36 months, while an accelerated weight and BMI increase was recorded in offspring of women with GDM in the first 6 months [84]. However, this study did not take into account the role of some confounders, such as preterm birth, adverse perinatal health outcomes, and severity of GDM, that may significantly affect infant growth.

In the “Exploring Perinatal Outcomes among Children (EPOCH)” study, the effect of BF on BMI curves has been retrospectively evaluated both in infants (0–26 months) and in children (27 months–13 years). From birth to 26 months, in the more intensive BF group, the mean BMI was significantly lower, and the BMI trajectory was slower compared with the low BF group only in infants born to mothers without GDM. In the childhood period, the effect of adequate BF on mean BMI and BMI curves became significant even in infants of GDM mothers after adjustment for sex, ethnicity, current childhood diet, and physical activity levels [85]. In the same cohort of infants, besides BMI, the effect of BF on adipose tissue distribution has been evaluated at 10 years and 16 years [86]. Although a non-significant increase in BMI and waist-to-hip ratio was observed, a history of GDM was associated with increased visceral adipose tissue, subcutaneous adipose tissue, and skinfold ratio in the low BF group. Differently, in the high BF group, only a significant increase in skinfold ratio was observed in infants born to GDM mothers. Of note, the worse adipose tissue distribution related to GDM history was counteracted by healthy behaviors, such as adequate diet and physical activity [86].

Aside from the role of well-known players, the beneficial effects of BF on long-term metabolic outcomes in children might be partly voided by the interference of emergent dietary modifiable factors, such as the high consumption of sugar-sweetened beverages in infancy. Indeed, most studies emphasized the role of sugar-rich beverage consumption as a key contributor to infant obesity and associated metabolic diseases [87]. The interaction between a history of GDM, BF, sugar-sweetened beverages consumption, and infant obesity has been evaluated in a recent study [88], showing that the positive effect of exclusive BF on the risk of developing obesity at 1–5 years of life in children born to mothers with prior GDM is neutralized by a high sugar-sweetened beverages intake. On the other hand, in the absence of GDM history, exclusive BF confers significant protection against infant obesity, independently of sugar-rich beverage consumption [88]. The same authors examined the relationship between GDM history, BF, and the prevalence of prediabetes and metabolic syndrome (MS) in childhood and adolescence (8–19 y). Specifically, the “Study of Latino Adolescents at Risk for Diabetes (SOLAR)” cohort involved *n* = 229 children, of whom *n* = 60 children had been exposed to GDM. Noteworthy, BF in children exposed to GDM was associated with an 18% and 10% lower risk of prediabetes and MS, respectively, compared with not breastfed children exposed to GDM [89].

Overall, a growing body of evidence from prospective studies has reported a connection between feeding patterns soon after delivery and the growth trajectories of offspring exposed to GDM in the intrauterine environment. In particular, in most studies, a high intensity and longer duration of BF were associated with slower infant growth curves compared with FF. Accelerated infant growth has been consistently linked to increased risk for future obesity, as well as exposure to GDM in the intrauterine environment. Thus, the beneficial effect of BF on growth trends might potentially counteract the heightened risk of obesity and metabolic diseases observed in infants born to mothers with GDM. Noteworthy, the favorable impact of BF on infant growth that had been previously described in offspring of mothers without GDM seems to extend to infants exposed to GDM. Importantly, in most studies, maternal factors such as sex and ethnicity and children’s lifestyle habits (diet and physical activity) did not significantly affect these results.

Thus, relevant evidence supports the beneficial role of BF on subsequent excessive adiposity in childhood even in the offspring of GDM mothers, although some limitations are to be mentioned. Noteworthy, the observational nature of most studies does not allow the establishment of a causal role. Accordingly, the impact of BF duration and intensity on infant obesity risk should be evaluated in specifically designed clinical trials. Indeed, one of the main limitations of the reported studies lies in the fact that, in most cases, information on BF patterns and lifestyle habits is collected once in the entire childhood period during the research visit. Furthermore, most studies did not consider the possible role of other confounders, such as socioeconomic factors, as well as maternal education, which might have a significant impact on diet quality after weaning. It should be also highlighted that the risk of obesity and T2D in children is multifactorial, involving genetic factors, socioeconomic status, food habits, and other environmental factors. Thus, the possible role of unknown confounders cannot be excluded, given that evidence largely emerges from prospective studies, not from randomized clinical trials.

## 5. Mechanisms Behind the Possible Beneficial Effects of BF Against Obesity and Metabolic Diseases in Offspring

Pregnancy and the early postnatal months are recognized as crucial periods of time in which the foundations for metabolic risk are laid. Observational evidence has highlighted that offspring of mothers with a history of GDM have consistently increased risk of overweight and obesity, thus being more susceptible to developing prediabetes, T2D, and MS later in life [13,90]. There are consistent data regarding the correlation between maternal lactation and offspring obesity. A longitudinal study involving over 150,000 children demonstrated a graded protective relationship between BF duration and the risk of overweight among non-Hispanic white subjects [91]. A meta-analysis of 10 studies showed that BF over the first 12 months of life decreased the odds of being overweight in childhood by 15% compared to exclusive FF infants [92]. Moreover, a meta-analysis of 23 studies, encompassing more than 1500 children, showed a pooled reduction of 13% in the prevalence of overweight or obesity [93]. Other data are consistent with these findings [94,95,96]. Overweight and obesity are a well-known risk factor for T2D. In a meta-analysis, a higher BMI in childhood was linked to an increased likelihood of diabetes in adulthood (OR 1.70; 95% CI 1.30–2.22) [97]. Therefore, a reduction in childhood overweight and obesity could be one of the protective mechanisms by which BF affects long-term infant health. However, a systematic review suggests that obesity is not a universal phenotype in children with T2D [98].

Therefore, further research is required to shed light on the role of BF and obesity on the pathogenesis of T2D in the young. The underlying reasons beyond the increased obesity risk in infants exposed to diabetes in the intrauterine milieu are not fully identified. An abnormal fat deposition, likely sustained by fetal hormonal alterations, such as high insulin and leptin levels, might significantly contribute to the disrupted metabolic health in infants born to mothers with previous GDM [22]. In addition, maternal hyperglycemia in the intrauterine environment may influence fetal gene expression in adipose tissue, leading to fat deposit enlargement and fetal metabolism perturbations [22].

In parallel, there is robust evidence that a sharp weight increase in the first months of life influences the risk of future overweight and obesity [80,99,100]. There is also consistent evidence that growth trajectories in the first 12 months are strictly linked to nutrition patterns. The benefits of BF against obesity risk in offspring are supported by robust evidence highlighting that intensive and long-lasting BF is linked to a more balanced infant growth trajectory, which prevents adipose tissue excess and dysfunction [101,102,103].

Overall, BF has been linked to a 13–22% reduced likelihood of obesity in future years [104]. The reasons behind the protective action of BF on obesity risk are not fully elucidated so far, but milk composition is thought to clearly influence neonatal growth patterns, indirectly favoring the onset of subsequent weight excess. Human milk is an extremely complex bio-fluid, whose constituents vary according to several factors, including the offspring’s needs, the stage of the lactation process, and maternal characteristics [16]. Although breastmilk substitutes are precious alternatives when BF is not feasible, formula milk composition largely differs from human milk both in the content and type of specific macronutrients, micronutrients, and bioactive compounds [105]. In particular, protein content, which is thought to be a crucial contributor to the accelerated weight increase in infants, is higher in formula milk than in breast milk [106,107]. There are hints that faster growth reported in formula-fed offspring might contribute to the metabolic imprinting of insulin production and secretion, leading to adipose tissue enlargement during the first 12 months of life [108]. Recently, there has been emerging interest in exploring the influence of the lipid composition of milk on growth patterns and, subsequently, on the development of metabolic diseases later in life. Specifically, human milk contains a consistent amount of specific fatty acids, such as alkyldiacylglcyerols and monoacylclycerides that might interfere with multiple mechanisms involved in growth processes, such as the modulation of the thermogenic activity of beige adipose tissue and the anti-inflammatory properties [109,110,111,112].

Recent scientific literature has elucidated the significant contribution of the gut microbiome to various health conditions. The development of the gut microbiome during early life appears to be a crucial factor in the onset of future diseases [113]. Multiple variables influence its formation, with BF emerging as a pivotal determinant. BF is associated with elevated levels of Bifidobacteria and Lactobacillus species, which exhibit more favorable health impacts compared to Escherichia coli, which is conversely reduced [114]. To illustrate this concept, a pilot study comprising 66 adult subjects demonstrated that individuals with T2D and obesity exhibited diminished levels of Bifidobacteria relative to healthy controls, lending support to the hypothesis that the microbiome significantly influences metabolic regulation [115]. In addition, a link between gut microbiota and infant growth has been reported in cohort studies [116]. Some components of human milk, including oligosaccharides, short-chain fatty acids (SCFAs), and antimicrobial proteins, have an impact on the infant gut microbiome, which in turn has been shown to influence body composition and growth patterns [116].

In light of the robust observational evidence reporting a protective effect of BF on excessive fetal growth and obesity risk [106,117], BF might counteract the detrimental effect of being exposed to GDM in utero. Although it is not yet clear whether BF has protective effects specifically on obesity risk in offspring exposed to GDM, the interest in this topic has considerably grown in the last years.

## 6. Conclusions and Future Perspectives

GDM is a frequent pregnancy complication whose adverse consequences are not limited to gestation and delivery. Long-term complications are fairly well defined, and the global increase in GDM prevalence urges for the adoption of cost-effective strategies to contrast the widespread obesity, T2D, and cardiovascular diseases both in mothers and infants.

A growing body of research has supported a possible beneficial influence of BF on glycolipid metabolism in women with a history of GDM in the early postpartum period. In particular, the vast majority of studies have shown a clear protective role of exclusive BF over FF or mixed feeding on glucose alterations at postpartum OGTT, with the prevalence of prediabetes and diabetes being therefore lower in the intensive BF groups compared to FF and mixed feeding in most studies. Interestingly, although attenuated, the effect of mixed feeding was significant over no BF groups, indicating that, when feasible, BF should be always encouraged to achieve better outcomes in the early postpartum period. Of note, these findings were even consistent over a long period of observation, and the graded association between BF duration and the entity of metabolic benefits, both in the short and in the long term, was consistent across studies.

BF appears to have a protective effect against the progression to T2D through multiple maternal mechanisms, not entirely elucidated. Key changes include enhanced glucose and lipid utilization, altered insulin sensitivity, and β-cell renewal. However, more research is needed to fully uncover these complex networks and their implications for maternal future health.

Although data are still evolving, it is believed that early infant feeding is a crucial determinant of future metabolic diseases. Specifically, rapid growth trajectories pose the basis for abnormal fat accumulation and dysregulation, thus increasing the risk for future obesity and cardiometabolic diseases.

The potential benefits of exclusive BF on mothers’ and infants’ metabolic health highlight the imperative for a global effort to overcome barriers to BF practice. A confidence gap among healthcare professionals, as well as the lack of well-structured supportive initiatives, might likely contribute to BF practice failure. The implementation of awareness about the inarguable benefits of BF on metabolic health among both physicians and patients, together with adequate communication strategies and supportive programs, are therefore mandatory to reduce the burden of GDM complications through cost-effective interventions.

## Data Availability

Not applicable.

## References

[1] American Diabetes Association Professional Practice Committee (2025). 2. Diagnosis and Classification of Diabetes: Standards of Care in Diabetes-2025. Diabetes Care.

[2] Zhang C., Rawal S., Chong Y.S. (2016). Risk factors for gestational diabetes: Is prevention possible?. Diabetologia.

[3] Schwartz N., Nachum Z., Green M.S. (2016). Risk factors of gestational diabetes mellitus recurrence: A meta-analysis. Endocrine.

[4] Kim C., Berger D.K., Chamany S. (2007). Recurrence of gestational diabetes mellitus: A systematic review. Diabetes Care.

[5] Bottalico J.N. (2007). Recurrent gestational diabetes: Risk factors, diagnosis, management, and implications. Semin. Perinatol..

[6] Vounzoulaki E., Khunti K., Abner S.C., Tan B.K., Davies M.J., Gillies C.L. (2020). Progression to type 2 diabetes in women with a known history of gestational diabetes: Systematic review and meta-analysis. BMJ.

[7] Rayanagoudar G., Hashi A.A., Zamora J., Khan K.S., Hitman G.A., Thangaratinam S. (2016). Quantification of the type 2 diabetes risk in women with gestational diabetes: A systematic review and meta-analysis of 95,750 women. Diabetologia.

[8] Xie W., Wang Y., Xiao S., Qiu L., Yu Y., Zhang Z. (2022). Association of gestational diabetes mellitus with overall and type specific cardiovascular and cerebrovascular diseases: Systematic review and meta-analysis. BMJ.

[9] Christensen M.H., Bistrup C., Rubin K.H., Nohr E.A., Vinter C.A., Andersen M.S., Moller S., Jensen D.M. (2024). Kidney Disease in Women With Previous Gestational Diabetes Mellitus: A Nationwide Register-Based Cohort Study. Diabetes Care.

[10] Metzger B.E., Lowe L.P., Dyer A.R., Trimble E.R., Chaovarindr U., Coustan D.R., Hadden D.R., McCance D.R., Hod M., HAPO Study Cooperative Research Group (2008). Hyperglycemia and adverse pregnancy outcomes. N. Engl. J. Med..

[11] Zhao P., Liu E., Qiao Y., Katzmarzyk P.T., Chaput J.P., Fogelholm M., Johnson W.D., Kuriyan R., Kurpad A., Lambert E.V. (2016). Maternal gestational diabetes and childhood obesity at age 9-11: Results of a multinational study. Diabetologia.

[12] Holder T., Giannini C., Santoro N., Pierpont B., Shaw M., Duran E., Caprio S., Weiss R. (2014). A low disposition index in adolescent offspring of mothers with gestational diabetes: A risk marker for the development of impaired glucose tolerance in youth. Diabetologia.

[13] Dabelea D., Mayer-Davis E.J., Lamichhane A.P., D’Agostino R.B., Liese A.D., Vehik K.S., Narayan K.M., Zeitler P., Hamman R.F. (2008). Association of intrauterine exposure to maternal diabetes and obesity with type 2 diabetes in youth: The SEARCH Case-Control Study. Diabetes Care.

[14] Clausen T.D., Mathiesen E.R., Hansen T., Pedersen O., Jensen D.M., Lauenborg J., Damm P. (2008). High prevalence of type 2 diabetes and pre-diabetes in adult offspring of women with gestational diabetes mellitus or type 1 diabetes: The role of intrauterine hyperglycemia. Diabetes Care.

[15] Li N., Yang Y., Cui D., Li C., Ma R.C.W., Li J., Yang X. (2021). Effects of lifestyle intervention on long-term risk of diabetes in women with prior gestational diabetes: A systematic review and meta-analysis of randomized controlled trials. Obes. Rev..

[16] Andreas N.J., Kampmann B., Mehring Le-Doare K. (2015). Human breast milk: A review on its composition and bioactivity. Early Hum. Dev..

[17] Patnode C.D., Henrikson N.B., Webber E.M., Blasi P.R., Senger C.A., Guirguis-Blake J.M. (2025). Breastfeeding and Health Outcomes for Infants and Children: A Systematic Review. Pediatrics.

[18] Frank N.M., Lynch K.F., Uusitalo U., Yang J., Lonnrot M., Virtanen S.M., Hyoty H., Norris J.M., for the TEDDY Study Group (2019). The relationship between breastfeeding and reported respiratory and gastrointestinal infection rates in young children. BMC Pediatr..

[19] Li W.J., Gao Y.C., Hu X., Tan Y.T., Deng J.J., Pan H.F., Tao S.S. (2025). Association between breastfeeding and the risk of autoimmune diseases: A systematic review and meta-analysis. Autoimmun. Rev..

[20] Blotsky A.L., Rahme E., Dahhou M., Nakhla M., Dasgupta K. (2019). Gestational diabetes associated with incident diabetes in childhood and youth: A retrospective cohort study. CMAJ.

[21] Dinleyici E.C. (2025). Breastfeeding and Health Benefits for the Mother-Infant Dyad: A Perspective on Human Milk Microbiota. Ann. Nutr. Metab..

[22] Meek J.Y., Noble L., Section on Breastfeeding (2022). Policy Statement: Breastfeeding and the Use of Human Milk. Pediatrics.

[23] World Health Organization Global Breastfeeding Scorecard 2023. Rates of Breastfeeding Increase Around the World Through Inproved Protection and Support. https://www.who.int/publications/i/item/WHO-HEP-NFS-23.17.

[24] Nguyen P.T.H., Pham N.M., Chu K.T., Van Duong D., Van Do D. (2019). Gestational Diabetes and Breastfeeding Outcomes: A Systematic Review. Asia Pac. J. Public Health.

[25] Catalano P.M., Shankar K. (2017). Obesity and pregnancy: Mechanisms of short term and long term adverse consequences for mother and child. BMJ.

[26] Wang Y., You H.X., Luo B.R. (2020). Exploring the breastfeeding knowledge level and its influencing factors of pregnant women with gestational diabetes mellitus. BMC Pregnancy Childbirth.

[27] Bellamy L., Casas J.P., Hingorani A.D., Williams D. (2009). Type 2 diabetes mellitus after gestational diabetes: A systematic review and meta-analysis. Lancet.

[28] Dennison R.A., Chen E.S., Green M.E., Legard C., Kotecha D., Farmer G., Sharp S.J., Ward R.J., Usher-Smith J.A., Griffin S.J. (2021). The absolute and relative risk of type 2 diabetes after gestational diabetes: A systematic review and meta-analysis of 129 studies. Diabetes Res. Clin. Pract..

[29] Kim C., Newton K.M., Knopp R.H. (2002). Gestational diabetes and the incidence of type 2 diabetes: A systematic review. Diabetes Care.

[30] de Gennaro G., Bianchi C., Aragona M., Battini L., Baronti W., Brocchi A., Del Prato S., Bertolotto A. (2020). Postpartum screening for type 2 diabetes mellitus in women with gestational diabetes: Is it really performed?. Diabetes Res. Clin. Pract..

[31] Brown S.D., Hedderson M.M., Zhu Y., Tsai A.L., Feng J., Quesenberry C.P., Ferrara A. (2022). Uptake of guideline-recommended postpartum diabetes screening among diverse women with gestational diabetes: Associations with patient factors in an integrated health system in USA. BMJ Open Diabetes Res. Care.

[32] Ogonowski J., Miazgowski T. (2009). The prevalence of 6 weeks postpartum abnormal glucose tolerance in Caucasian women with gestational diabetes. Diabetes Res. Clin. Pract..

[33] O’Reilly M.W., Avalos G., Dennedy M.C., O’Sullivan E.P., Dunne F. (2011). Atlantic DIP: High prevalence of abnormal glucose tolerance post partum is reduced by breast-feeding in women with prior gestational diabetes mellitus. Eur. J. Endocrinol..

[34] Bianchi C., de Gennaro G., Brocchi A., Minaldi E., Del Prato S., Bertolotto A. (2021). Risk factors associated with postpartum impaired glucose regulation in women with previous gestational diabetes. J. Diabetes Complicat..

[35] Arnoriaga-Rodriguez M., Melero V., Barabash A., Valerio J., Del Valle L., O’Connor R.M., de Miguel P., Diaz J.A., Familiar C., Moraga I. (2023). Modifiable Risk Factors and Trends in Changes in Glucose Regulation during the First Three Years Postdelivery: The St Carlos Gestational Diabetes Mellitus Prevention Cohort. Nutrients.

[36] Gunderson E.P., Hedderson M.M., Chiang V., Crites Y., Walton D., Azevedo R.A., Fox G., Elmasian C., Young S., Salvador N. (2012). Lactation intensity and postpartum maternal glucose tolerance and insulin resistance in women with recent GDM: The SWIFT cohort. Diabetes Care.

[37] Gunderson E.P., Kim C., Quesenberry C.P., Marcovina S., Walton D., Azevedo R.A., Fox G., Elmasian C., Young S., Salvador N. (2014). Lactation intensity and fasting plasma lipids, lipoproteins, non-esterified free fatty acids, leptin and adiponectin in postpartum women with recent gestational diabetes mellitus: The SWIFT cohort. Metabolism.

[38] Vanlaer Y., Minschart C., Vrolijk H., Van Crombrugge P., Moyson C., Verhaeghe J., Devlieger R., Vandeginste S., Verlaenen H., Vercammen C. (2024). Impact of breastfeeding on risk of glucose intolerance in early postpartum after gestational diabetes. Front. Endocrinol..

[39] Hebeisen I., Gonzalez Rodriguez E., Arhab A., Gross J., Schenk S., Gilbert L., Benhalima K., Horsch A., Quansah D.Y., Puder J.J. (2024). Prospective associations between breast feeding, metabolic health, inflammation and bone density in women with prior gestational diabetes mellitus. BMJ Open Diabetes Res. Care.

[40] Shub A., Miranda M., Georgiou H.M., McCarthy E.A., Lappas M. (2019). The effect of breastfeeding on postpartum glucose tolerance and lipid profiles in women with gestational diabetes mellitus. Int. Breastfeed. J..

[41] Yasuhi I., Yamashita H., Maeda K., Nomiyama M., Mizunoe T., Tada K., Yorozu M., Ogawa M., Kodama T., Yamaguchi K. (2019). High-intensity breastfeeding improves insulin sensitivity during early post-partum period in obese women with gestational diabetes. Diabetes Metab. Res. Rev..

[42] Corrado F., Giunta L., Granese R., Corrado S., Micali M., Santamaria A., D’Anna R., Di Benedetto A. (2019). Metabolic effects of breastfeeding in women with previous gestational diabetes diagnosed according to the IADPSG criteria. J. Matern. Fetal Neonatal Med..

[43] Suthasmalee S., Phaloprakarn C. (2024). Lactation duration and development of type 2 diabetes and metabolic syndrome in postpartum women with recent gestational diabetes mellitus. Int. Breastfeed. J..

[44] Zhang Z., Lai M., Piro A.L., Alexeeff S.E., Allalou A., Rost H.L., Dai F.F., Wheeler M.B., Gunderson E.P. (2021). Intensive lactation among women with recent gestational diabetes significantly alters the early postpartum circulating lipid profile: The SWIFT study. BMC Med..

[45] Funai K., Lodhi I.J., Spears L.D., Yin L., Song H., Klein S., Semenkovich C.F. (2016). Skeletal Muscle Phospholipid Metabolism Regulates Insulin Sensitivity and Contractile Function. Diabetes.

[46] Zuarez-Easton S., Berkovich I., Birenbaum-Carmeli D., Tal A., Zoabi R., Salim R. (2020). Effect of lactation on the recurrence rate of gestational diabetes mellitus: A retrospective cohort study. Arch. Gynecol. Obstet..

[47] Melov S.J., White L., Simmons M., Kirby A., Stulz V., Padmanabhan S., Alahakoon T.I., Pasupathy D., Cheung N.W. (2022). The BLIiNG study—Breastfeeding length and intensity in gestational diabetes and metabolic effects in a subsequent pregnancy: A cohort study. Midwifery.

[48] Melov S.J., Elhindi J., White L., McNab J., Lee V.W., Donnolley K., Alahakoon T.I., Padmanabhan S., Cheung N.W., Pasupathy D. (2023). Previous High-Intensity Breastfeeding Lowers the Risk of an Abnormal Fasting Glucose in a Subsequent Pregnancy Oral Glucose Tolerance Test. Nutrients.

[49] Gunderson E.P., Hurston S.R., Ning X., Lo J.C., Crites Y., Walton D., Dewey K.G., Azevedo R.A., Young S., Fox G. (2015). Lactation and Progression to Type 2 Diabetes Mellitus After Gestational Diabetes Mellitus: A Prospective Cohort Study. Ann. Intern. Med..

[50] O’Shea E., Awang M.H., Kgosidialwa O., Tuthill A. (2023). Abnormal glucose tolerance in women with prior gestational diabetes mellitus: A 4-year follow-up study. Ir. J. Med. Sci..

[51] Hewage S.S., Koh X.Y.H., Soh S.E., Pang W.W., Fok D., Cai S., Muller-Riemenschneider F., Yap F., Tan K.H., Chua M.C. (2021). Breastfeeding Duration and Development of Dysglycemia in Women Who Had Gestational Diabetes Mellitus: Evidence from the GUSTO Cohort Study. Nutrients.

[52] Feleke B.E., Feleke T.E., Kassahun M.B., Adane W.G., Achenefe D., Genetu A., Nigussie A.A., Engedaw H.A. (2021). Progression of pregnancy induced diabetes mellitus to type two diabetes mellitus, an ambidirectional cohort study. Prim. Care Diabetes.

[53] Gunderson E.P., Lewis C.E., Lin Y., Sorel M., Gross M., Sidney S., Jacobs D.R., Shikany J.M., Quesenberry C.P. (2018). Lactation Duration and Progression to Diabetes in Women Across the Childbearing Years: The 30-Year CARDIA Study. JAMA Intern. Med..

[54] Ziegler A.G., Wallner M., Kaiser I., Rossbauer M., Harsunen M.H., Lachmann L., Maier J., Winkler C., Hummel S. (2012). Long-term protective effect of lactation on the development of type 2 diabetes in women with recent gestational diabetes mellitus. Diabetes.

[55] Wander P.L., Hinkle S.N., Enquobahrie D.A., Wu J., Ley S.H., Grunnet L.G., Chavarro J.E., Li M., Bjerregaard A.A., Liu A. (2022). Cumulative Lactation and Clinical Metabolic Outcomes at Mid-Life among Women with a History of Gestational Diabetes. Nutrients.

[56] Stuebe A.M., Rich-Edwards J.W., Willett W.C., Manson J.E., Michels K.B. (2005). Duration of lactation and incidence of type 2 diabetes. JAMA.

[57] Ley S.H., Chavarro J.E., Li M., Bao W., Hinkle S.N., Wander P.L., Rich-Edwards J., Olsen S., Vaag A., Damm P. (2020). Lactation Duration and Long-term Risk for Incident Type 2 Diabetes in Women With a History of Gestational Diabetes Mellitus. Diabetes Care.

[58] Ma S., Hu S., Liang H., Xiao Y., Tan H. (2019). Metabolic effects of breastfeed in women with prior gestational diabetes mellitus: A systematic review and meta-analysis. Diabetes Metab. Res. Rev..

[59] Anhe G.F., Bordin S. (2022). The adaptation of maternal energy metabolism to lactation and its underlying mechanisms. Mol. Cell Endocrinol..

[60] Burnol A.F., Leturque A., Ferre P., Kande J., Girard J. (1986). Increased insulin sensitivity and responsiveness during lactation in rats. Am. J. Physiol..

[61] Jones R.G., Ilic V., Williamson D.H. (1984). Physiological significance of altered insulin metabolism in the conscious rat during lactation. Biochem. J..

[62] Burnol A.F., Loizeau M., Girard J. (1990). Insulin receptor activity and insulin sensitivity in mammary gland of lactating rats. Am. J. Physiol..

[63] Butte N.F., Hopkinson J.M., Mehta N., Moon J.K., Smith E.O. (1999). Adjustments in energy expenditure and substrate utilization during late pregnancy and lactation. Am. J. Clin. Nutr..

[64] Gunderson E.P., Crites Y., Chiang V., Walton D., Azevedo R.A., Fox G., Elmasian C., Young S., Salvador N., Lum M. (2012). Influence of breastfeeding during the postpartum oral glucose tolerance test on plasma glucose and insulin. Obstet. Gynecol..

[65] Xiang A.H., Kawakubo M., Kjos S.L., Buchanan T.A. (2006). Long-acting injectable progestin contraception and risk of type 2 diabetes in Latino women with prior gestational diabetes mellitus. Diabetes Care.

[66] Monroy G., Fernandez C., Caballe T., Altimira L., Corcoy R. (2022). Breastfeeding effect on glucose tolerance assessment in women with previous gestational diabetes mellitus: A randomized controlled trial. Diabet. Med..

[67] Sanderson M., O’Hara H., Foderingham N., Dupont W.D., Shu X.O., Peterson N., Fair A.M., Disher A.C. (2015). Type 2 diabetes and mammographic breast density among underserved women. Cancer Causes Control.

[68] Ken-Dror G., Fluck D., Lean M.E.J., Casanueva F.F., Han T.S. (2024). The relationship between low prolactin and type 2 diabetes. Rev. Endocr. Metab. Disord..

[69] Retnakaran R., Ye C., Kramer C.K., Connelly P.W., Hanley A.J., Sermer M., Zinman B. (2016). Maternal Serum Prolactin and Prediction of Postpartum beta-Cell Function and Risk of Prediabetes/Diabetes. Diabetes Care.

[70] Zhang Z., Piro A.L., Allalou A., Alexeeff S.E., Dai F.F., Gunderson E.P., Wheeler M.B. (2022). Prolactin and Maternal Metabolism in Women With a Recent GDM Pregnancy and Links to Future T2D: The SWIFT Study. J. Clin. Endocrinol. Metab..

[71] Vasavada R.C., Gonzalez-Pertusa J.A., Fujinaka Y., Fiaschi-Taesch N., Cozar-Castellano I., Garcia-Ocana A. (2006). Growth factors and beta cell replication. Int. J. Biochem. Cell Biol..

[72] Freemark M., Avril I., Fleenor D., Driscoll P., Petro A., Opara E., Kendall W., Oden J., Bridges S., Binart N. (2002). Targeted deletion of the PRL receptor: Effects on islet development, insulin production, and glucose tolerance. Endocrinology.

[73] Ben-Jonathan N., Hugo E.R., Brandebourg T.D., LaPensee C.R. (2006). Focus on prolactin as a metabolic hormone. Trends Endocrinol. Metab..

[74] Bensadoun A. (1991). Lipoprotein lipase. Annu. Rev. Nutr..

[75] McNestry C., Crowley R.K., O’Reilly S.L., Kasemiire A., Callanan S., Delahunt A., Twomey P.J., McAuliffe F.M. (2024). Breastfeeding duration is associated with favorable body composition and lower glycoprotein acetyls in later life. Int. J. Gynaecol. Obstet..

[76] Ramos-Roman M.A., Syed-Abdul M.M., Casey B.M., Alger J.R., Liu Y.L., Parks E.J. (2022). Lactation alters the relationship between liver lipid synthesis and hepatic fat stores in the postpartum period. J. Lipid Res..

[77] Bril F., Barb D., Portillo-Sanchez P., Biernacki D., Lomonaco R., Suman A., Weber M.H., Budd J.T., Lupi M.E., Cusi K. (2017). Metabolic and histological implications of intrahepatic triglyceride content in nonalcoholic fatty liver disease. Hepatology.

[78] Ruiz-Otero N., Tessem J.S., Banerjee R.R. (2024). Pancreatic islet adaptation in pregnancy and postpartum. Trends Endocrinol. Metab..

[79] Moon J.H., Kim H., Kim H., Park J., Choi W., Choi W., Hong H.J., Ro H.J., Jun S., Choi S.H. (2020). Lactation improves pancreatic beta cell mass and function through serotonin production. Sci. Transl. Med..

[80] Plagemann A., Harder T., Rodekamp E., Kohlhoff R. (2012). Rapid neonatal weight gain increases risk of childhood overweight in offspring of diabetic mothers. J. Perinat. Med..

[81] Fenger-Gron J., Fenger-Gron M., Blunck C.H., Schonemann-Rigel H., Wielandt H.B. (2015). Low breastfeeding rates and body mass index in Danish children of women with gestational diabetes mellitus. Int. Breastfeed. J..

[82] Gunderson E.P., Greenspan L.C., Faith M.S., Hurston S.R., Quesenberry C.P., Investigators S.O.S. (2018). Breastfeeding and growth during infancy among offspring of mothers with gestational diabetes mellitus: A prospective cohort study. Pediatr. Obes..

[83] Longmore D.K., Titmuss A., Barr E., Barzi F., Simmonds A., Lee I.L., Hawthorne E., Derkenne R., Connors C., Boyle J. (2022). Breastfeeding and infant growth in offspring of mothers with hyperglycaemia in pregnancy: The pregnancy and neonatal diabetes outcomes in remote Australia study. Pediatr. Obes..

[84] Aris I.M., Soh S.E., Tint M.T., Saw S.M., Rajadurai V.S., Godfrey K.M., Gluckman P.D., Yap F., Chong Y.S., Lee Y.S. (2017). Associations of infant milk feed type on early postnatal growth of offspring exposed and unexposed to gestational diabetes in utero. Eur. J. Nutr..

[85] Crume T.L., Ogden L.G., Mayer-Davis E.J., Hamman R.F., Norris J.M., Bischoff K.J., McDuffie R., Dabelea D. (2012). The impact of neonatal breast-feeding on growth trajectories of youth exposed and unexposed to diabetes in utero: The EPOCH Study. Int. J. Obes..

[86] Sauder K.A., Bekelman T.A., Harrall K.K., Glueck D.H., Dabelea D. (2019). Gestational diabetes exposure and adiposity outcomes in childhood and adolescence: An analysis of effect modification by breastfeeding, diet quality, and physical activity in the EPOCH study. Pediatr. Obes..

[87] Cantoral A., Tellez-Rojo M.M., Ettinger A.S., Hu H., Hernandez-Avila M., Peterson K. (2016). Early introduction and cumulative consumption of sugar-sweetened beverages during the pre-school period and risk of obesity at 8–14 years of age. Pediatr. Obes..

[88] Vandyousefi S., Whaley S.E., Widen E.M., Asigbee F.M., Landry M.J., Ghaddar R., Davis J.N. (2019). Association of breastfeeding and early exposure to sugar-sweetened beverages with obesity prevalence in offspring born to mothers with and without gestational diabetes mellitus. Pediatr. Obes..

[89] Vandyousefi S., Goran M.I., Gunderson E.P., Khazaee E., Landry M.J., Ghaddar R., Asigbee F.M., Davis J.N. (2019). Association of breastfeeding and gestational diabetes mellitus with the prevalence of prediabetes and the metabolic syndrome in offspring of Hispanic mothers. Pediatr. Obes..

[90] Davis J.N., Gunderson E.P., Gyllenhammer L.E., Goran M.I. (2013). Impact of gestational diabetes mellitus on pubertal changes in adiposity and metabolic profiles in Latino offspring. J. Pediatr..

[91] Grummer-Strawn L.M., Mei Z., Centers for Disease C., Prevention Pediatric Nutrition Surveillance S. (2004). Does breastfeeding protect against pediatric overweight? Analysis of longitudinal data from the Centers for Disease Control and Prevention Pediatric Nutrition Surveillance System. Pediatrics.

[92] Weng S.F., Redsell S.A., Swift J.A., Yang M., Glazebrook C.P. (2012). Systematic review and meta-analyses of risk factors for childhood overweight identifiable during infancy. Arch. Dis. Child..

[93] Victora C.G., Bahl R., Barros A.J., Franca G.V., Horton S., Krasevec J., Murch S., Sankar M.J., Walker N., Rollins N.C. (2016). Breastfeeding in the 21st century: Epidemiology, mechanisms, and lifelong effect. Lancet.

[94] Yan J., Liu L., Zhu Y., Huang G., Wang P.P. (2014). The association between breastfeeding and childhood obesity: A meta-analysis. BMC Public Health.

[95] Rito A.I., Buoncristiano M., Spinelli A., Salanave B., Kunesova M., Hejgaard T., Garcia Solano M., Fijalkowska A., Sturua L., Hyska J. (2019). Association between Characteristics at Birth, Breastfeeding and Obesity in 22 Countries: The WHO European Childhood Obesity Surveillance Initiative—COSI 2015/2017. Obes. Facts.

[96] Horta B.L., Rollins N., Dias M.S., Garcez V., Perez-Escamilla R. (2023). Systematic review and meta-analysis of breastfeeding and later overweight or obesity expands on previous study for World Health Organization. Acta Paediatr..

[97] Llewellyn A., Simmonds M., Owen C.G., Woolacott N. (2016). Childhood obesity as a predictor of morbidity in adulthood: A systematic review and meta-analysis. Obes. Rev..

[98] Cioana M., Deng J., Nadarajah A., Hou M., Qiu Y., Chen S.S.J., Rivas A., Banfield L., Toor P.P., Zhou F. (2022). The Prevalence of Obesity Among Children With Type 2 Diabetes: A Systematic Review and Meta-analysis. JAMA Netw. Open.

[99] Ekelund U., Ong K., Linne Y., Neovius M., Brage S., Dunger D.B., Wareham N.J., Rossner S. (2006). Upward weight percentile crossing in infancy and early childhood independently predicts fat mass in young adults: The Stockholm Weight Development Study (SWEDES). Am. J. Clin. Nutr..

[100] Taveras E.M., Rifas-Shiman S.L., Belfort M.B., Kleinman K.P., Oken E., Gillman M.W. (2009). Weight status in the first 6 months of life and obesity at 3 years of age. Pediatrics.

[101] Dewey K.G. (2003). Is breastfeeding protective against child obesity?. J. Hum. Lact..

[102] Durmus B., Heppe D.H., Gishti O., Manniesing R., Abrahamse-Berkeveld M., van der Beek E.M., Hofman A., Duijts L., Gaillard R., Jaddoe V.W. (2014). General and abdominal fat outcomes in school-age children associated with infant breastfeeding patterns. Am. J. Clin. Nutr..

[103] Owen C.G., Martin R.M., Whincup P.H., Smith G.D., Cook D.G. (2005). Effect of infant feeding on the risk of obesity across the life course: A quantitative review of published evidence. Pediatrics.

[104] Arenz S., Ruckerl R., Koletzko B., von Kries R. (2004). Breast-feeding and childhood obesity—A systematic review. Int. J. Obes. Relat. Metab. Disord..

[105] Tang M. (2018). Protein Intake during the First Two Years of Life and Its Association with Growth and Risk of Overweight. Int. J. Environ. Res. Public Health.

[106] Kramer M.S., Guo T., Platt R.W., Vanilovich I., Sevkovskaya Z., Dzikovich I., Michaelsen K.F., Dewey K., Promotion of Breastfeeding Intervention Trials Study, G (2004). Feeding effects on growth during infancy. J. Pediatr..

[107] Heinig M.J., Nommsen L.A., Peerson J.M., Lonnerdal B., Dewey K.G. (1993). Energy and protein intakes of breast-fed and formula-fed infants during the first year of life and their association with growth velocity: The DARLING Study. Am. J. Clin. Nutr..

[108] Ong K.K., Petry C.J., Emmett P.M., Sandhu M.S., Kiess W., Hales C.N., Ness A.R., Dunger D.B., the ALSPAC Study Team (2004). Insulin sensitivity and secretion in normal children related to size at birth, postnatal growth, and plasma insulin-like growth factor-I levels. Diabetologia.

[109] Yu H., Dilbaz S., Cossmann J., Hoang A.C., Diedrich V., Herwig A., Harauma A., Hoshi Y., Moriguchi T., Landgraf K. (2019). Breast milk alkylglycerols sustain beige adipocytes through adipose tissue macrophages. J. Clin. Investig..

[110] Isaacs C.E., Kashyap S., Heird W.C., Thormar H. (1990). Antiviral and antibacterial lipids in human milk and infant formula feeds. Arch. Dis. Child..

[111] Hellmuth C., Uhl O., Demmelmair H., Grunewald M., Auricchio R., Castillejo G., Korponay-Szabo I.R., Polanco I., Roca M., Vriezinga S.L. (2018). The impact of human breast milk components on the infant metabolism. PLoS ONE.

[112] George A.D., Burugupalli S., Paul S., Mansell T., Burgner D., Meikle P.J. (2022). The Role of Human Milk Lipids and Lipid Metabolites in Protecting the Infant against Non-Communicable Disease. Int. J. Mol. Sci..

[113] Stiemsma L.T., Michels K.B. (2018). The Role of the Microbiome in the Developmental Origins of Health and Disease. Pediatrics.

[114] Suarez-Martinez C., Santaella-Pascual M., Yague-Guirao G., Martinez-Gracia C. (2023). Infant gut microbiota colonization: Influence of prenatal and postnatal factors, focusing on diet. Front. Microbiol..

[115] Sroka-Oleksiak A., Mlodzinska A., Bulanda M., Salamon D., Major P., Stanek M., Gosiewski T. (2020). Metagenomic Analysis of Duodenal Microbiota Reveals a Potential Biomarker of Dysbiosis in the Course of Obesity and Type 2 Diabetes: A Pilot Study. J. Clin. Med..

[116] Ma J., Palmer D.J., Geddes D., Lai C.T., Stinson L. (2022). Human Milk Microbiome and Microbiome-Related Products: Potential Modulators of Infant Growth. Nutrients.

[117] Stettler N., Stallings V.A., Troxel A.B., Zhao J., Schinnar R., Nelson S.E., Ziegler E.E., Strom B.L. (2005). Weight gain in the first week of life and overweight in adulthood: A cohort study of European American subjects fed infant formula. Circulation.

